# MicroRNA-Dependent Transcriptional Silencing of Transposable Elements in Drosophila Follicle Cells

**DOI:** 10.1371/journal.pgen.1005194

**Published:** 2015-05-19

**Authors:** Bruno Mugat, Abdou Akkouche, Vincent Serrano, Claudia Armenise, Blaise Li, Christine Brun, Tudor A. Fulga, David Van Vactor, Alain Pélisson, Séverine Chambeyron

**Affiliations:** 1 Institut de Génétique Humaine, Centre National de la Recherche Scientifique, Montpellier, France; 2 Department of Cell Biology and Program in Neuroscience, Harvard Medical School, Boston, Massachusetts, United States of America; University of Cambridge, UNITED KINGDOM

## Abstract

RNA interference-related silencing mechanisms concern very diverse and distinct biological processes, from gene regulation (*via* the microRNA pathway) to defense against molecular parasites (through the small interfering RNA and the Piwi-interacting RNA pathways). Small non-coding RNAs serve as specificity factors that guide effector proteins to ribonucleic acid targets *via* base-pairing interactions, to achieve transcriptional or post-transcriptional regulation. Because of the small sequence complementarity required for microRNA-dependent post-transcriptional regulation, thousands of microRNA (miRNA) putative targets have been annotated in Drosophila. In Drosophila somatic ovarian cells, genomic parasites, such as transposable elements (TEs), are transcriptionally repressed by chromatin changes induced by Piwi-interacting RNAs (piRNAs) that prevent them from invading the germinal genome. Here we show, for the first time, that a functional miRNA pathway is required for the piRNA-mediated transcriptional silencing of TEs in this tissue. Global miRNA depletion, caused by tissue- and stage-specific knock down of *drosha* (involved in miRNA biogenesis), *AGO1* or *gawky* (both responsible for miRNA activity), resulted in loss of TE-derived piRNAs and chromatin-mediated transcriptional de-silencing of TEs. This specific TE de-repression was also observed upon individual titration (by expression of the complementary miRNA sponge) of two miRNAs (miR-14 and miR-34) as well as in a miR-14 loss-of-function mutant background. Interestingly, the miRNA defects differentially affected TE- and 3' UTR-derived piRNAs. To our knowledge, this is the first indication of possible differences in the biogenesis or stability of TE- and 3' UTR-derived piRNAs. This work is one of the examples of detectable phenotypes caused by loss of individual miRNAs in *Drosophila* and the first genetic evidence that miRNAs have a role in the maintenance of genome stability *via* piRNA-mediated TE repression.

## Author Summary

The fine-tuning of gene expression required for the normal development of multicellular organisms involves small RNAs that are called microRNAs (miRNAs). MiRNAs can reduce the stability or the activity of the many cellular messenger RNAs that contain miRNA complementary sequences. In animal gonads, the harmful expression and proliferation of genomic parasites, such as transposable elements, is prevented by a similar, sequence homology-based silencing mechanism that involves a different class of small RNAs, the Piwi-interacting RNAs (piRNAs). We report here that, in Drosophila somatic ovarian tissues, two miRNAs, miR-14 and miR-34, are required for the accumulation of piRNAs that prevent the expression of transposable elements and, probably, the subsequent invasion of the germinal genome. On the other hand, we found that other sources of piRNA production, such as the 3' end of genes, are miRNA-independent, suggesting the existence of variations in the piRNA biogenesis pathways depending on the piRNA genomic origin. Our results therefore highlight a novel miRNA function in the maintenance of genome stability through piRNA-mediated TE repression.

## Introduction

In many, if not most, eukaryotes, RNA silencing is responsible for the regulation of gene expression *via* the association of small, 20–30 nucleotide (nt)-long, non-coding RNAs with Argonaute proteins (reviews: [1–4]). Partial or perfect base pairing between the small RNAs and their RNA targets provides the specificity for the repressive activities of the Argonaute-containing effector complexes, called RNA-induced silencing complexes (RISCs). In *Drosophila melanogaster*, the Argonaute protein family includes two clades: the AGO proteins (AGO1 and AGO2) and the PIWI proteins (Piwi, Aubergine (Aub) and Argonaute 3 (AGO3)). Each RISC contains one of three types of small regulatory RNAs that have different roles and mechanisms of action. Specifically, more than 230 AGO1-associated microRNAs (miRNAs; 21- to 23 nt in length) regulate gene expression, during development (reviews: [5,6]. On the other hand, AGO2-associated small-interfering RNAs (siRNAs; 21 nt-long), and PIWI-interacting RNAs (piRNAs; 23 to 30 nt in length) are more dedicated to the defence against exogenous and endogenous parasites, such as viruses and transposable elements (TEs) [7–11].

Argonaute-mediated silencing can occur at the transcriptional or post-transcriptional level. Most Argonaute proteins, such as siRNA-loaded AGO2 [12] or piRNA-loaded Aub [13], are endowed with endo-nucleolytic slicer activity that is required for their post-transcriptional gene silencing (PTGS) function through direct cleavage of RNA targets. In contrast, slicer-independent miRNA-mediated PTGS usually occurs through the association of AGO1 with GW182 (also called Gawky), leading to mRNA translation inhibition and destabilization [14,15]. The slicer activity is conserved in Piwi, but does not seem to be required for its silencing function [16,17]. Indeed, piRNA-loaded Piwi guides the deposition of repressive chromatin marks on TE sequences resulting in their transcriptional silencing [18–23].

In *Drosophila* adult ovarian somatic support cells (follicle cells), piRNA-mediated TE transcriptional repression is exclusively achieved by the loading onto Piwi of primary piRNAs generated by unidirectional transcription of heterochromatic loci, called piRNA clusters, such as *flamenco* [24]. A number of coding genes also give rise to piRNAs from their 3’ untranslated regions (3’UTRs) [25]. In follicle cells, the *traffic jam* (*tj*), *jim* and *GC32000* genes are the major producers of 3’UTR-derived piRNAs [26]. The role of this class of Piwi-loaded genic piRNAs is still elusive. Although TE- and 3’UTR-derived piRNAs originate from different genomic loci, they seem to use the same biogenesis pathway, because 3'UTR-derived piRNAs are affected by defects in all the proteins known to be involved in the biogenesis of TE-derived piRNAs [17,27,28]. Two recent genetic screens have highlighted the complexity of the somatic ovarian piRNA pathway that involves many proteins with different gene ontologies [29,30]. However, except Gawky, none of the proteins that are directly involved in the miRNA pathway were identified by these screens [29].

We show here that piRNA-mediated TE transcriptional repression is impaired in follicle cells in which the miRNA pathway is defective following Drosha, Gawky or AGO1 inactivation. Moreover, we report that individual titration of two miRNAs (miR-14 and miR-34) leads to a similar TE de-repression phenotype. New germinal insertions of retroviral-like TEs can result from their somatic ovarian expression [31,32]. Therefore, these findings provide the first genetic evidence that loss of miRNA function could impair maintenance of genome stability *via* TE de-repression. Moreover, differently from what observed for TE-derived piRNAs, accumulation of 3'UTR-derived piRNAs was not affected by the same defects in the miRNA pathway, highlighting unsuspected differences between these piRNA pathways.

## Results

### TE de-silencing in Drosophila follicle cells in which the miRNA pathway is defective

To test whether miRNAs are required for TE repression in Drosophila follicle cells, we impaired the miRNA pathway by expressing either double stranded RNAs (RNAi) or a dominant negative mutant construct under the control of the tissue-specific driver *traffic jam Gal4* (*tj-*GAL4) [27,33]. To preserve the essential miRNA roles during early development, we restricted miRNA depletion (thereafter called "soma KD") to the adult stage (follicle cells) by transiently inactivating the *Gal80*^ts^ thermo-sensitive Gal4 inhibitor [34]. After a shift at 25°C for five days, TE desilencing was monitored in follicle cells using the *ZAM*-lacZ reporter transgene [35].

**Fig 1:**
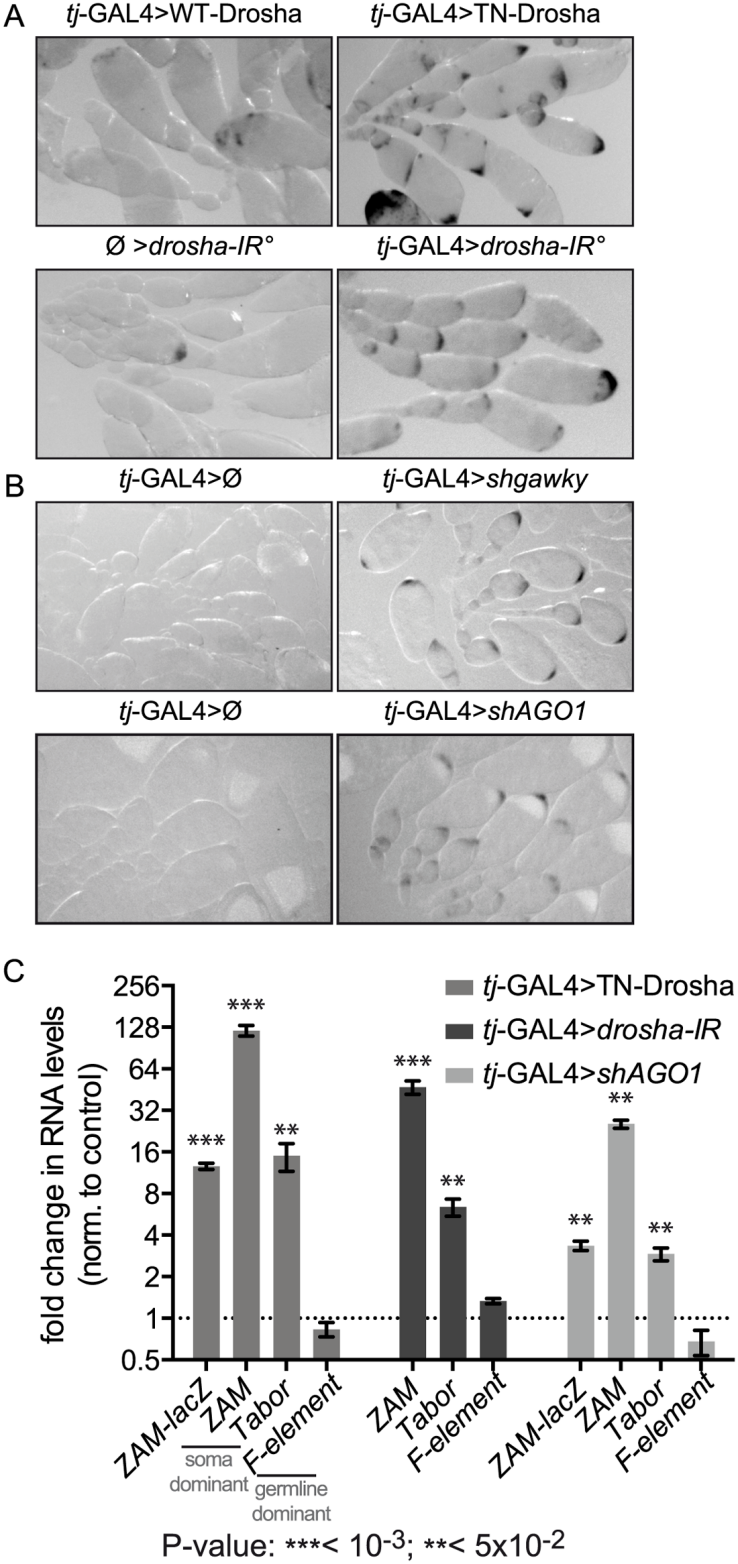
Follicle cell-specific defects in the miRNA pathway lead to *ZAM-*lacZ reporter and somatic TE de-repression. (A) Comparison of *ZAM*-lacZ reporter expression in control ovaries in which WT-Drosha expression is driven by the *tj-*GAL4 somatic driver (*tj*-GAL4>WT-Drosha) or that contain two independent hairpins against Drosha without any driver (Ø>*drosha-IR°*) and in ovaries in which *tj-*GAL4 drives the expression of the trans-dominant negative Drosha construct (*tj*-GAL4>TN-Drosha) or the two Drosha long hairpins (*tj*-GAL4>*drosha-IR°*). The blue β-Gal staining is shown in black. (B) Comparison of *ZAM-*lacZ reporter expression in ovaries where *gawky* (*tj*-GAL4>sh*gawky*) or *AGO1* (*tj-GAL4*>sh*AGO1*) were silenced by *tj*-GAL4-induced shRNA expression, and in ovaries from the respective sibling controls (*tj*-GAL4>Ø). The blue β-Gal staining is shown in black. (C) Fold changes in the steady-state RNA levels of the *ZAM*-lacZ reporter (PCR1 primer pair, S3 Table, Fig 2A), the somatic TEs *ZAM* and *Tabor* and the germline-specific TE *F-element*, following the expression, in follicle cells, of the trans-dominant negative Drosha construct (*tj*-GAL4>TN-Drosha), the *Drosha* long hairpins (*tj*-GAL4>*drosha-IR*) or the *AGO1* small hairpin (*tj*-GAL4>*shAGO1*). In *tj*-GAL4>Drosha-IR ovaries, the *ZAM*-lacZ reporter was replaced by the *UAS-dcr2* transgene (see S2 Table). Quantification was done relative to *RpL32* and normalized to the respective controls (*tj*-GAL4>Ø, Ø>*drosha-IR* and *tj*-GAL4>*shAGO3*) (error bars represent the standard deviation (S.D.) of three biological replicates, log2 scale).

We first impaired miRNA biogenesis, by targeting the Drosha protein. Indeed, Drosha functions as the catalytic subunit of the Microprocessor complex that initiates miRNA production [36]. To achieve efficient RNAi against Drosha in follicle cells, we had to co-express the Dicer-2 RNAi enhancer with two long hairpins against Drosha (S1 and S2 Tables). We also constructed and expressed a trans-dominant negative Drosha mutant (TN-Drosha), which contains a point mutation in each RNAseIII domain, and a wild-type Drosha construct (WT-Drosha) as control (see Materials and Methods, S1 Text and S1A Fig). As the TN-Drosha mutant had been previously used to impair miRNA production only in cell culture [37], we first checked whether this trans-dominant negative approach was effective also in Drosophila follicle cells (S1 Fig). Expression of TN-Drosha in follicle cells resulted in the formation of an inactive Microprocessor complex that could not process pri-miRNAs (S1B and S1C Fig) leading to a detectable depletion of miRNAs (S1D Fig). We then monitored TE repression after having impaired Drosha function in follicle cells either by RNAi (*tj*-GAL4>*drosha-IR*) or by using the trans-dominant negative approach (*tj*-GAL4>TN-Drosha). We found that the *ZAM*-lacZ reporter activity was de-repressed in the posterior follicular epithelium compared to cells expressing the respective negative controls (Ø>*drosha-IR*; *tj*-GAL4>WT-Drosha) (Fig 1A).

To test whether the whole miRNA pathway is required for TE repression, we also knocked down Gawky and AGO1, two proteins of the miRNA effector complex, by short hairpin(sh)-mediated RNAi [38]. Again, we observed tissue-specific β-Gal staining only in ovaries in which *Gawky* or *AGO1* was knocked down specifically in follicle cells (*tj*-GAL4>*shgawky* and *tj*-GAL4>*shAGO1* respectively), indicating de-repression of the *ZAM*-lacZ reporter activity (Fig 1B). Thus, the effector complex of the miRNA pathway seems to be essential also for somatic TE repression.

To quantify the extent of reporter de-repression and to investigate whether somatic endogenous TEs were also de-repressed, we determined by quantitative RT-PCR the steady-state RNA levels of the lacZ reporter transgene and of members of two TE families (*ZAM* and *Tabor*) that are specifically repressed in follicle cells (Fig 1C). In addition to the transcripts of the *ZAM*-lacZ reporter transgene, transcripts of the *ZAM* and *Tabor* TE families accumulated significantly upon follicle cell-specific impairment of Drosha and AGO1. Conversely, the expression of the *F-element*, a TE specifically repressed in the germline (negative control), was not affected by inactivation of the miRNA pathway in follicle cells (Fig 1C). Therefore, our data indicate that the repression of the *ZAM-*lacZ reporter transgene and of two TE families, repressed specifically in follicle cells, is miRNA-dependent.

### The miRNA pathway is required for chromatin-mediated TE transcriptional silencing in Drosophila follicle cells

**Fig 2:**
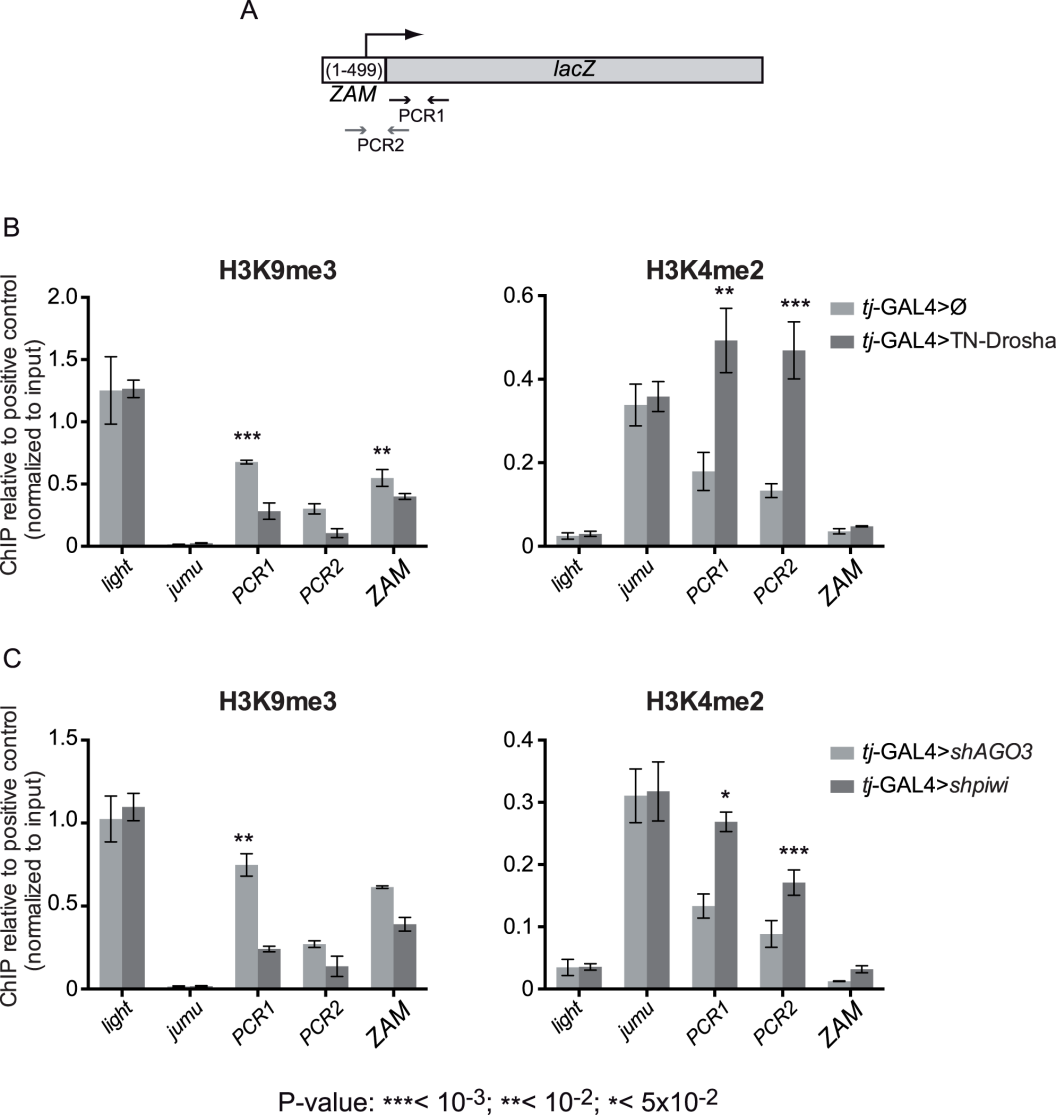
Evidence for miRNA-dependent transcriptional silencing of a follicle cell reporter transgene and an endogenous somatic TE. (A) Cartoon showing the *ZAM* region included in the *ZAM*-lacZ reporter construct and the localization of the PCR-1 and PCR-2 primer pairs (arrows and S3 Table). (B) ChIP-qPCR using ovarian chromatin to monitor the effects of *tj-*driven expression of the trans-dominant negative Drosha construct (*tj*-GAL4>TN-Drosha) (dark grey bars). Control ovaries express the somatic driver alone (*tj*-GAL4>Ø) (light grey bars). (C) ChIP-qPCR using ovarian chromatin to monitor the effects of *tj-*driven *piwi* soma KD (dark grey bars). Control ovaries were from *AGO3* soma KD flies because AGO3 is not expressed in follicle cells (light grey bars). H3K9me3 enrichment was quantified relative to the *1360-element* (positive control) and normalized to input. *light* and *jumu* are examples of actively transcribed genes in heterochromatic and euchromatic regions, respectively. H3K4me2 enrichment was quantified relative to *RpL15* (positive control) and normalized to input. *light* was used as a negative control and *jumu* as an example of actively transcribed euchromatic gene (bars represent the mean ± SD of ≥3 biological replicates); *P-*values were determined using the two-tailed Student’s t-test, when series had a normal distribution, or the Mann-Whitney test.

We then checked whether the TE de-repression observed upon impairment of the miRNA pathway occurred at the transcriptional level. Indeed, in follicle cells, Piwi-dependent TE transcriptional silencing is associated with the presence of the Histone H3 lysine 9 trimethylation (H3K9me3) "repressive mark" and the absence of the Histone H3 lysine 4 dimethylation (H3K4me2) "active mark" on active TE copies [21,39]. As the chromatin immunoprecipitation-quantitative polymerase chain reaction (ChIP-qPCR) technique does not always discriminate between active euchromatic and defective heterochromatic copies of a TE family [40], we focused our study on two regions of the single-copy *ZAM*-lacZ reporter transgene (Fig 2A: PCR1 and PCR2).

We studied the chromatin changes on this reporter transgene upon *tj-*driven expression of TN-Drosha in follicle cells (Fig 2B). The absence of Drosha activity resulted in the increase of H3K4me2 and the decrease of H3K9me3 marks on the transgene. We also observed a significant H3K9me3 decrease by using the *ZAM* primer pair that detects members of the *ZAM* TE family (Fig 2B, left panel).

To compare the chromatin changes observed upon *tj-*driven expression of TN-Drosha with the chromatin changes caused by Piwi depletion, we studied, by ChIP-qPCR, the chromatin of the *ZAM*-lacZ transgene upon *tj-*driven *piwi* soma KD. As AGO3 is not expressed in this tissue, we used *AGO3* soma KD as negative control (Fig 2C). TN-Drosha expression and the *piwi* somatic knockdown resulted in comparable H3K4me2 increase and H3K9me3 decrease on the *ZAM*-lacZ transgene.

These findings suggest that the miRNA pathway is required in follicle cells for the piRNA-mediated transcriptional silencing of retrotransposons.

### Specific loss of the TE-targeting piRNAs in Drosophila follicle cells lacking Drosha activity

**Fig 3:**
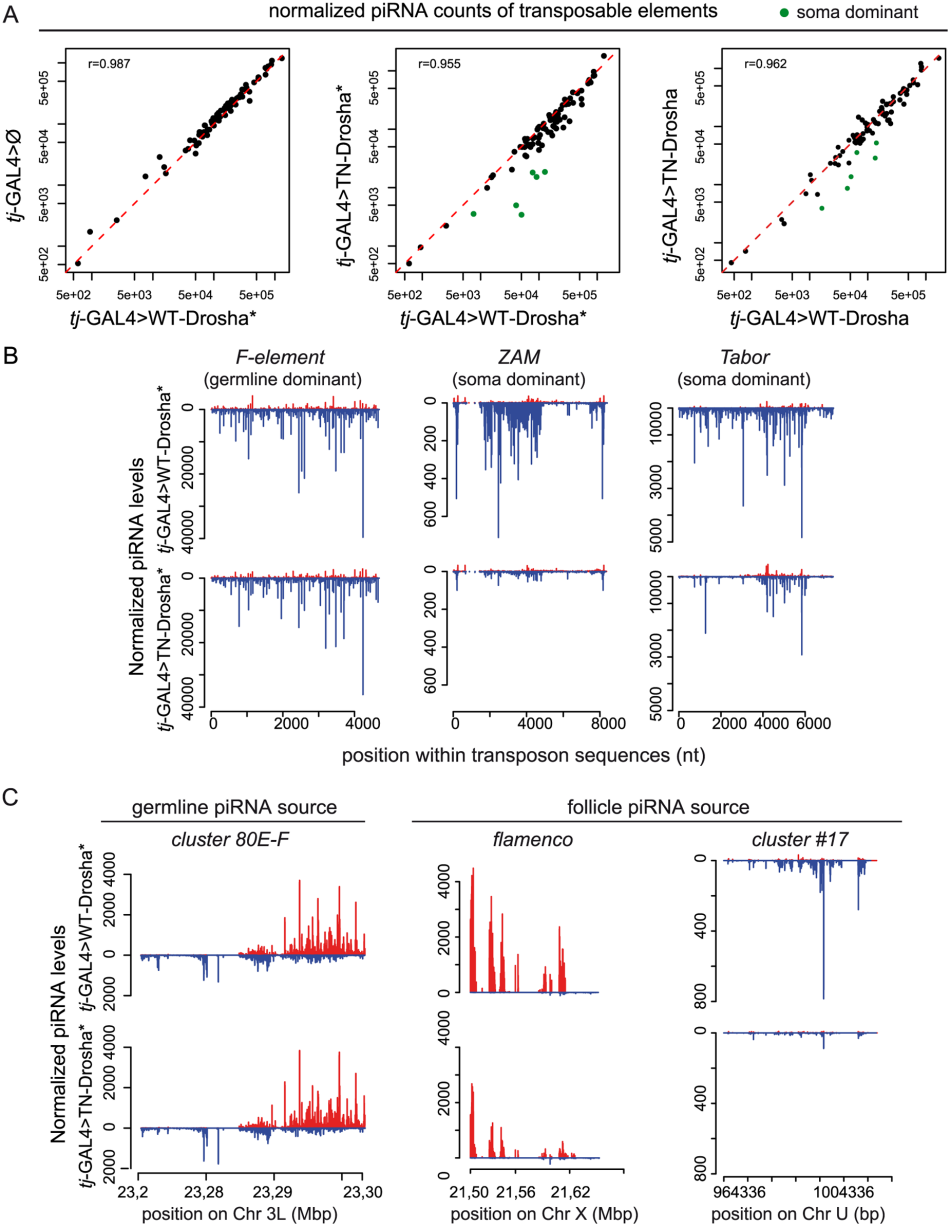
Loss of TE-derived piRNAs in Drosophila follicle cells that express the trans-dominant negative Drosha construct. (A) Scatter plots show the correlation between the normalized piRNA abundance for each of the 85 most targeted *Drosophila* TEs (up to four mismatches allowed between reads and RepBase sequence). The Pearson correlation [r] was based on all TEs. piRNA counts were compared pairwise between libraries from transgenic ovaries that contain *tj-*driven WT-Drosha (*x* axes), the *tj*-driver-alone (*tj*-GAL4>Ø) (*y* axis, left panel) or *tj-*driven TN-Drosha (*y* axes, middle and right panels). The asterisks indicate that the corresponding small RNA libraries were made using ovaries that do not contain the *tubP-Gal80*^*ts*^ transgene (see S2 Table). Libraries were normalized to 1 million of piRNAs that map uniquely to 42AB, a germline-specific piRNA cluster. The six follicle cell-specific (soma dominant) TEs (green dots in the middle and right panels) were identified by Malone et al 2009. (B-C) Normalized profiles of ovarian piRNAs (sense: up (red); antisense: down (blue)) that map to TE consensus sequences or piRNA clusters in *tj*-GAL4>WT-Drosha* (upper panels) and *tj*-GAL4>TN-Drosha* (lower panels) libraries. The y axis indicates the number of piRNAs the 5’ end of which matches the x axis sequence at a given position. (B) Profiles of piRNAs mapping to the *F-element*, a germline-specific TE (left), or to *ZAM* (middle) and *Tabor* (right), two soma-dominant TEs. (C) Profiles of piRNAs that originate from 80E-F, a germline-specific piRNA cluster (left), or from *flamenco* (middle) and cluster # 17(right), two follicle cell-specific piRNA clusters (only genome-unique piRNAs are profiled).

To determine whether specific piRNA populations were affected upon miRNA depletion, we sequenced and analysed total ovarian small RNA (18 to 29 nt) libraries from five genetic backgrounds shifted at 25°C for five days (S1 and S2 Tables). We prepared the first library using ovaries containing the driver alone *(tj-*GAL4>Ø). In two other libraries (annotated with asterisks in Fig 3), we combined the driver with the TN-Drosha or the WT-Drosha control transgene. The last two libraries (Fig 3A and S3 Fig) were replicates of the two previous ones, except that we used ovaries containing the conditional *tub-Gal80*^ts^ thermo-sensitive Gal4 inhibitor (S1 and S2 Tables). We normalized the libraries to 1 million of piRNAs produced by the 42AB germline-specific piRNA cluster. Fig 3A shows the number of piRNA reads mapping to each of the 85 most targeted Drosophila TEs [41]. First, we observed that expression of WT-Drosha had no effect on the piRNA populations, because the number of piRNAs targeting each TE family was comparable in the *tj-*GAL4>Ø and *tj-*GAL4>WT-Drosha libraries (Fig 3A). Therefore, we used ovaries that express WT-Drosha in follicle cells as controls to compare the effect of TN-Drosha expression in the same cells.

In two independent experiments, we observed a decrease of piRNA reads for the soma-dominant TEs (*i*.*e*., the most highly targeted TEs in this tissue) [41], in both *tj-*GAL4>TN-Drosha libraries compared to the respective *tj-*GAL4>WT-Drosha control libraries (green dots in Fig 3A). Particularly, in both *tj-*GAL4>TN-Drosha libraries, the number of antisense piRNAs mapping across the *ZAM* and *Tabor* sequences (two examples of soma-dominant TEs) was reduced, whereas piRNAs targeting germline-dominant TEs, such as the *F-element*, were not affected (Fig 3B and S3A Fig). In follicle cells, piRNAs are mainly produced by a soma-specific piRNA cluster called *flamenco*. Differently from germline-specific piRNA clusters, such as cluster 42AB (used as a normalizer in this study) and 80EF, *flamenco* piRNAs were four times less abundant in the *tj-*GAL4>TN-Drosha* than in the *tj-*GAL4*>*WT-Drosha* library (Fig 3C).

**Fig 4:**
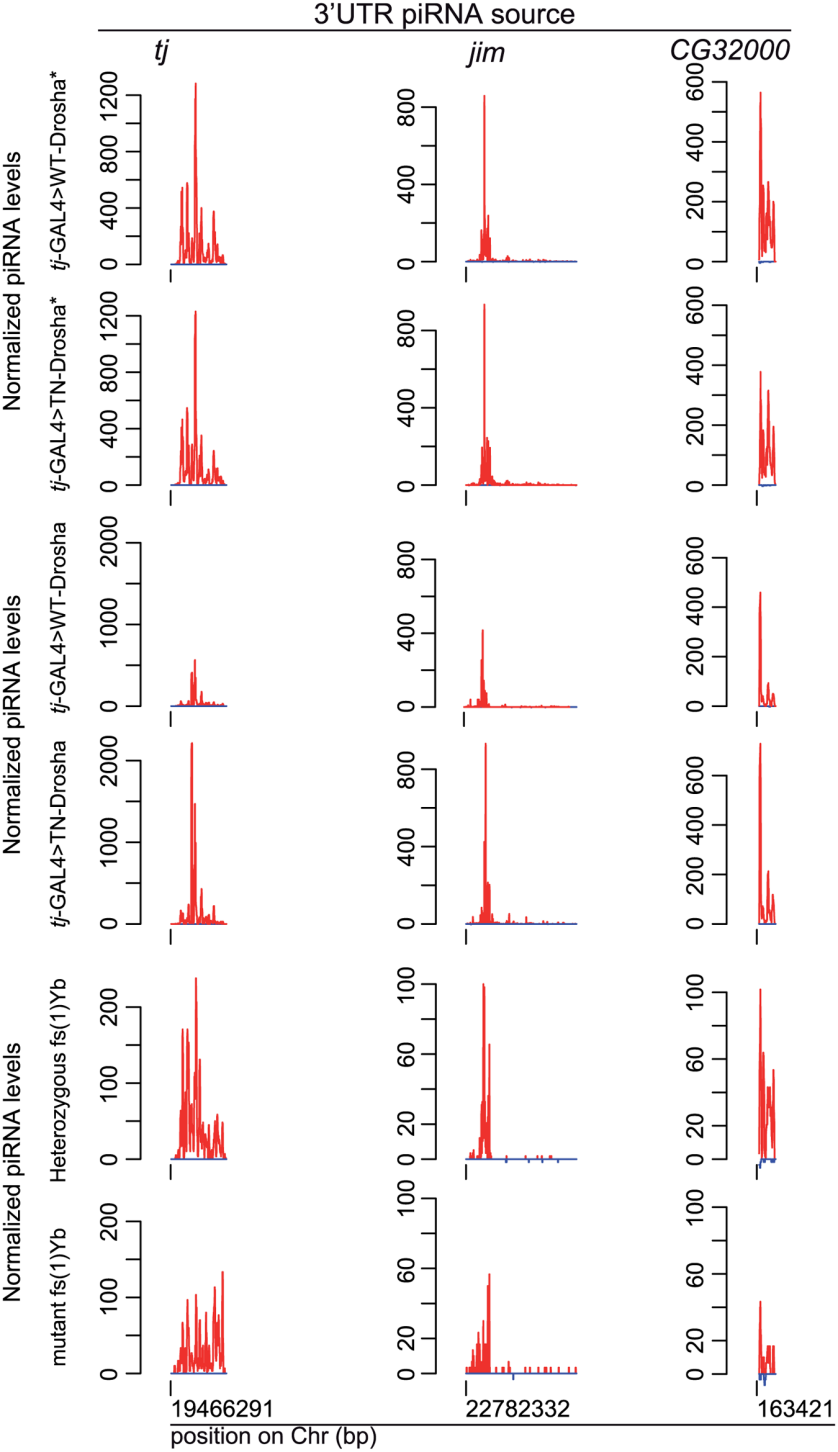
The amount of 3’UTR-derived piRNAs does not decrease in follicle cells lacking Drosha activity. Normalized profiles of ovarian piRNAs (sense: up (red); antisense: down (blue)) that map to the *traffic jam*, *jim* and *GC32000* 3'UTRs in *tj*-GAL4>WT-Drosha* and *tj*-GAL4>TN-Drosha* libraries (upper panels), in *tj*-GAL4>WT-Drosha and *tj*-GAL4>TN-Drosha (middle panels) and in *fs(1)Yb* heterozygous and homozygous libraries (lower panels). The y axis indicates the number of piRNAs the 5’ end of which matches, at a given position, the x axis sequence corresponding to the whole 3'UTR. Only genome-unique piRNAs were profiled.

Surprisingly, accumulation of piRNAs produced by the 3’ untranslated region of the *traffic jam* (*tj*) gene did not seem to be affected by miRNA depletion in both TN-Drosha libraries (Fig 4). This was also true for *jim* and *CG32000*, two other genes that produce abundant piRNAs in ovarian somatic cultured cells (Fig 4) [26]. The 3’UTR piRNA profile seemed therefore to be unaffected in TN-Drosha libraries. Conversely, the previously described Yb mutant libraries showed a general reduction of all classes of ovarian somatic piRNAs (Fig 4) [42].

**Fig 5:**
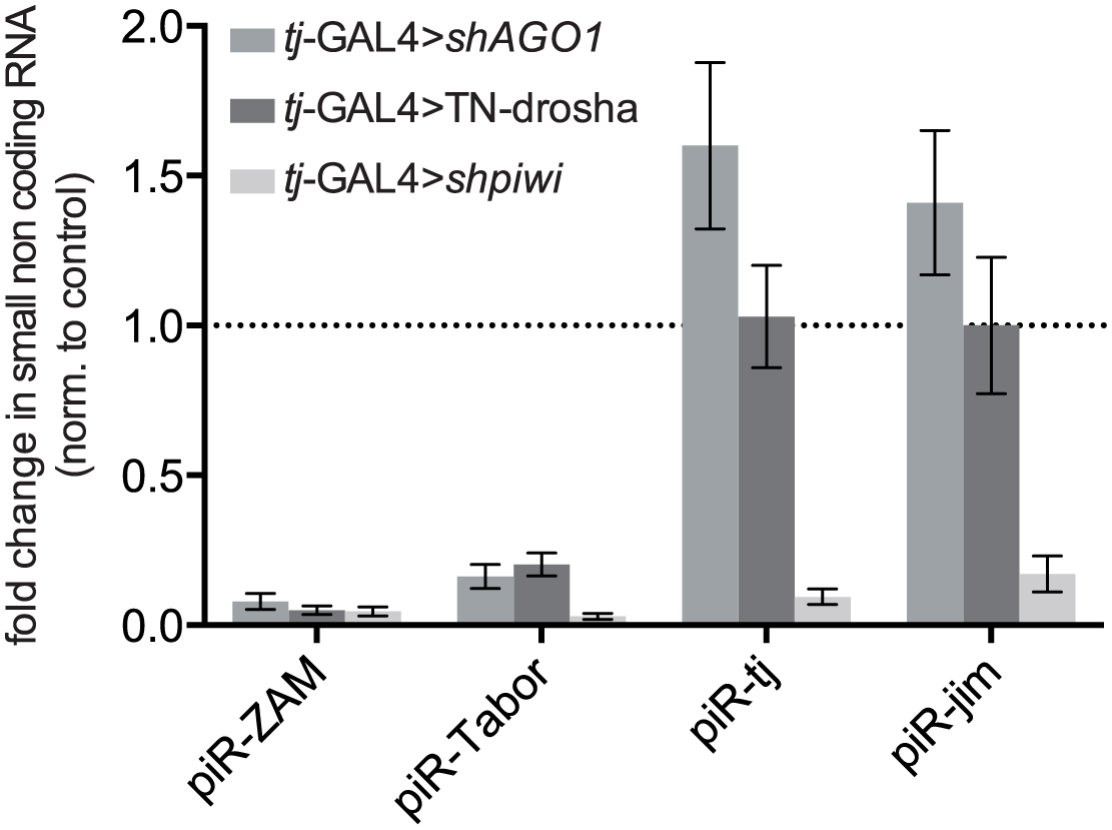
Effect of the impairment of the piRNA and miRNA pathways on the accumulation of four individual piRNAs. Analysis by RT-qPCR of the changes in the steady-state levels of four major piRNAs originating from *ZAM* and *Tabor* (two follicle cell-specific TEs) and from the 3' UTR of the *traffic jam* (*tj*) and *jim* genes (see sequences in S3 Table), upon *AGO1* soma KD (*tj*-GAL4>*shAGO1*), expression of the trans-dominant negative Drosha construct (*tj*-GAL4>TN-Drosha), or *piwi* soma KD (*tj*-GAL4>*shpiwi*). Quantification was done relative to the 42AB piRNA (a germline-specific piRNA: S3 Table) and normalized to the respective controls (*tj*-GAL4>sh*AGO3*, *tj*-GAL4>Ø and *tj*-GAL4>sh*AGO3*) (bars represent the mean ± SD of ≥ three biological replicates). P-values were evaluated by two-tailed Student’s t-tests (for *ZAM* and *Tabor*, all P-values were lower than 0.007).

To monitor individual piRNAs without the need of high throughput sequencing, we adapted a procedure for miRNA quantification [43] to quantify individual small RNAs by RT-qPCR (see S1 Text and S3 Table). Using this method, we could demonstrate that, like in *tj-*GAL4>TN-Drosha ovaries, two major *ZAM* and *Tabor* antisense piRNAs were also significantly depleted in ovaries upon *AGO1* soma-KD, whereas the amounts of two major 3’UTR piRNAs (*tj* and *jim)* were unaffected (Fig 5). Altogether, our observations suggest that, in follicle cells, accumulation of TE-targeting piRNAs is specifically dependent on the activity of the miRNA pathway.

### A miR-sponge screen of miRNAs required for piRNA-mediated TE silencing

**Fig 6:**
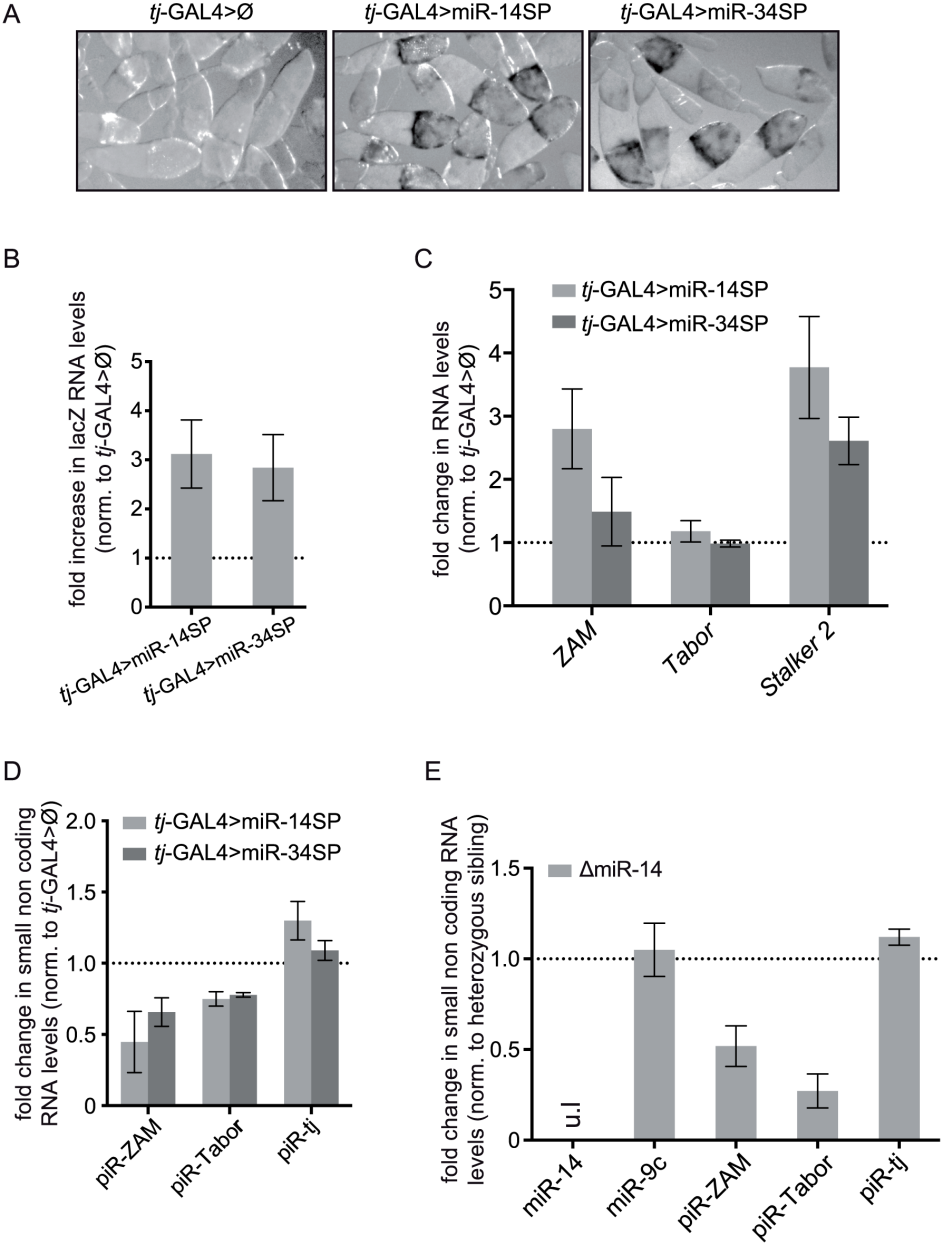
miRNA screen and genetic validation of the requirement of miR-14 for TE repression. (A) Detection of the piRNA sensor expression following titration of two positive miRNAs by *tj-*driven expression of the corresponding miRNA-sponges, miR-14SP (middle) and miR-34SP (right). After 1h of β-Gal staining no staining was observed in the sibling ovaries without any miRNA-sponge (Ø), as illustrated by the control for the miR-14SP experiment (left). At that time, only de-repression of the *gypsy-*lacZ reporter gene, but not yet of the *ZAM-*lacZ reporter gene, could be detected. (B) Fold changes in the steady-state RNA levels of the lacZ reporters (see primer sequence in S3 Table) upon miR-14SP- and miR-34SP-induced miRNA titration. Quantification was done relative to *RpL32* and normalized to sibling ovaries with no miRNA sponge (error bars represent ± SD; n = three biological replicates). (C) Fold changes in the steady-state RNA levels of three follicle cell-specific TEs (*ZAM*, *Tabor* and *Stalker2*) upon miR-14SP and miR-34SP *tj*-driven expression. Quantification was done relative to RpL32 and normalized to sibling ovaries with no miRNA-sponge (error bars represent the SD of three biological replicates). The absence of *Tabor* de-repression might indicate that the *Tabor* family lacks active elements in the tested genotypes. (D) Fold changes in the steady-state level of the three major *ZAM*, *Tabor* and *traffic jam* (*tj*) piRNAs (see sequences in S3 Table), upon miR-14SP- and miR-34SP-induced miRNA titration. Quantification was done relative to miR-9c and normalized to sibling ovaries with no miRNA sponge (error bars represent the S.D. of three biological replicates). (E) Fold changes in miRNA and piRNA levels induced by the miR-14 null mutation (ΔmiR-14). Quantification was done relative to miR-989 and normalized to heterozygous sibling ovaries. ul: undetectable level (error bars represent the SD of three biological replicates).

As our results indicated that TE de-repression is caused by follicle cell-specific general miRNA depletion, we then screened individual Drosophila miRNAs to identify which miRNA(s) is (are) essential for TE regulation. Around 230 miRNAs have been annotated in *Drosophila*. To determine which miRNAs are effectively expressed in follicle cells, we took advantage of the inability of TN-Drosha to cleave its pri-miRNA targets that, therefore, remain strongly bound to it. By immunoprecipitation of RNA bound to TN-Drosha in *tj-*GAL4>TN-Drosha ovarian extracts (Materials and Methods), we identified a subset of 53 Drosha-dependent miRNAs expressed in this tissue (S5 Table). We could then test 47 of these miRNAs using second generation miRNA-sponges (miR-SP). These sponges allow the titration of a given miRNA by tissue-specific over-expression of a non-coding RNA containing 20 binding sites for that miRNA [44,45]. Each of the 47 miR-SP constructs was expressed by two *tj*-driven autosomal transgenes, in the presence of two TE repression reporters (*gypsy*-lacZ and *ZAM-*lacZ). MiR-SP mediated titration of two miRNAs (miR-14 and miR-34) resulted in lacZ de-repression, as indicated by β-Gal staining and RT-qPCR (Fig 6A–6B). After 1h of staining, we observed only the *gypsy*-lacZ pattern, in agreement with the fact that the *ZAM-*lacZ reporter has got a much lower expression level (see Materials and Methods). We also detected de-repression of endogenous copies of three other somatic TE families (*ZAM*, *Tabor and Stalker2*) (Fig 6C).

Moreover, like upon *drosha* and *AGO1* knock down, the level of two piRNAs (*ZAM* and *Tabor*), quantified by RT-qPCR, was clearly decreased following miR-SP-induced miR-14 and miR-34 titration (Fig 6D). We could partly confirm the results of this screen by using a miR-14 null mutant. The presence of a comparable piRNA loss (Fig 6E) and TE de-repression (S4 Fig), in this mutant ruled out a possible off-target effect of the miR-14-SP approach. Two Drosha-dependent miRNAs, miR-14 and miR-34, are therefore individually required for both TE repression and TE-derived piRNA accumulation in follicle cells.

### Looking for miRNA target(s) involved in the piRNA pathway

Next we wanted to identify the gene(s) that are regulated by miR-14 and miR-34 for piRNA-mediated TE repression in follicle cells. Using the Targetscan miRNA target predictor (http://www.targetscan.org/fly_12/), we found 153 and 98 putative targets for miR-14 and miR-34, respectively. As depletion of these miRNAs leads to up-regulation of their targets and TE-derived piRNA collapse, these target genes should be considered as inhibitors of the piRNA pathway. This may explain why none of them corresponded to any of the many hits of two previous screens performed to identify genes required for piRNA-mediated TE repression [29,30].

**Fig 7:**
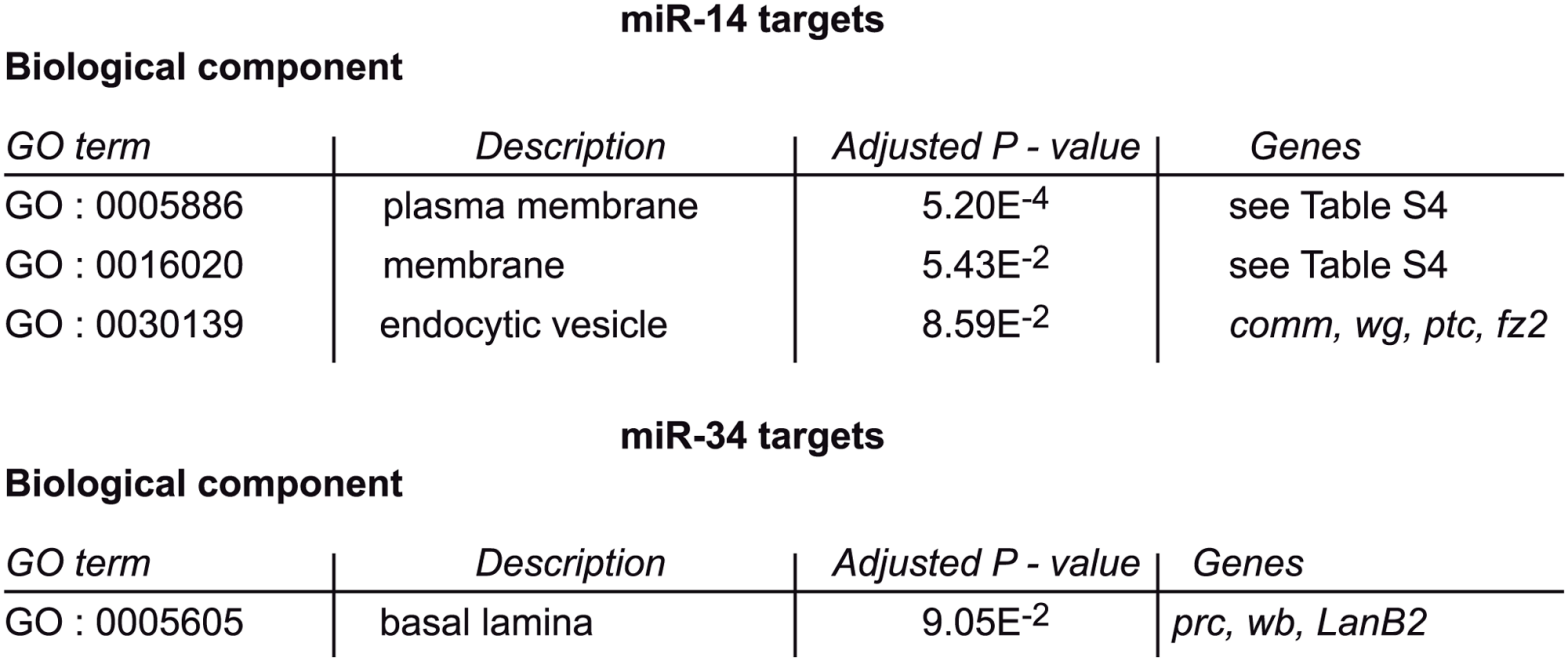
Plasma membrane signaling might be involved in miRNA-dependent TE repression. GO term comparisons of miR-14 and miR-34 putative targets and of all the Drosophila miRNA putative targets. The list of genes included in GO terms 0005886 and 0016020 is in S6 Table.

To determine whether these putative inhibitors of the piRNA pathway are part of a single biological process that antagonizes piRNA-mediated TE repression, we performed a gene ontology (GO) term enrichment analysis on the miR-14 and miR-34 target genes using GOrilla (http://cbl-gorilla.cs.technion.ac.il/). We compared the 153 miR-14 and the 98 miR-34 putative targets using as background set the 3759 genes that are putatively targeted by all Drosophila miRNAs. The miR-34 target genes only overlapped modestly with the GO term “basal lamina component”. Conversely, the “plasma membrane component” GO term was significantly enriched in miR-14 target genes (P-value 5.2E-4) (Fig 7 and S6 Table). This observation might be related to the hypothesis that a transmembrane signalling pathway is involved in somatic ovarian TE repression [46].

Alternatively, a piRNA pathway modifier might be an indirect miRNA target controlled by a regulatory cascade downstream of a direct miRNA target gene. The effect of miRNAs on their direct targets is usually very small as the reduction of the mRNA level is no more than 2-fold [47,48]. By contrast, taking advantage of the inputs of the RIP-seq experiments to compare transcriptomes, we noted that, upon miRNA depletion in follicle cells, most changes corresponded to more than 2-fold increase or to a decrease of the total ovarian steady-state RNA level (S7 Table). It is technically very difficult to determine how many of these indirect target genes are actual effectors of piRNA-dependent repression. For this reason we did not try to identify the direct or indirect miRNA target genes (either agonists or antagonists of the piRNA pathway) that are responsible for the observed miRNA-dependent TE repression.

### Drosha endonuclease activity is not directly required for piRNA biosynthesis

We then asked whether Drosha could be directly involved in nuclear processing of piRNA precursors, in addition to its indirect effect on piRNAs *via* miRNA biogenesis. Indeed, we hypothesized that piRNA cluster transcripts, which are likely to fold into many hairpins because of their length and repetitive content, could be putative Drosha substrates. To test this theory, we first quantified the steady state level of RNAs derived from the *flamenco* locus in control and tj-Gal4>TN-Drosha ovaries. By quantitative RT-PCR using primer pairs spanning five different regions of *flamenco* [49] (S3 Table), we found no evidence that *flamenco*-derived long RNAs accumulated in tj-Gal4>TN-Drosha ovaries (S5 Fig). Moreover, in RNAs co-immunoprecipitated with the TN-Drosha protein we did not observe any enrichment of reads mapping to *flamenco* as it was the case for the reads mapping to pri-miRNAs (S4 Table). Considered as a whole, these data strongly suggest that Drosha endonucleolytic activity is not directly involved in the production of primary piRNAs from the *flamenco* locus.

## Discussion

### Loss of a single miRNA is sufficient to impair piRNA-mediated TE repression in follicle cells

Recent advances from genetic and genomic studies have highlighted the importance of miRNAs in many aspects of animal development such as cell proliferation, differentiation, morphogenesis and apoptosis [50,51]. For instance, in *Drosophila*, oogenesis requires the miRNA pathway in both follicle and germ cells [52–57].

We show here that in follicle cells with defective miRNA function (by knocking down effectors of the miRNA pathway, such as *AGO1* and *Gawky*) or biogenesis (through *drosha* knock down or expression of a dominant negative Drosha protein), TE-derived piRNA levels are strongly reduced and piRNA-mediated transcriptional TE repression is impaired. We observed similar phenotypes upon individual titration (by expression of the corresponding miR-sponge) of miR-14 and miR-34, and also in a miR-14 loss of function mutant. As retroviral-like TEs need to be expressed in the somatic ovarian tissue to invade the germinal genome, our data add maintenance of genome integrity, *via* piRNA-mediated TE repression, to the list of miRNA-controlled biological functions.

### piRNA loss does not seem to result from change in follicle cell fate

As *tj-*driven constitutive knockdown of the miRNA pathway affected ovarian morphology, we considered the possibility that piRNA impairment could be caused by the loss of follicle cell fate. However, the following four observations do not support this hypothesis: (1) Based on the finding that the steady state level of *flamenco* transcripts was unaffected (S5 Fig), this hypothetical cell fate change would not result in the lack of piRNA precursors due to the overall reduction of tissue-specific transcription of piRNA clusters. (2) Even in distorted ovaries where the vitellogenic oocyte was no longer located at the posterior end of the egg chamber, the *ZAM*-lacZ reporter was always derepressed in the area of the follicular epithelium facing the vitellus (S6 Fig). This was reminiscent of the normal tissue-specific ZAM expression pattern, suggesting that miRNA depletion did not affect cell fate to such an extent as to prevent EGF-receptor signaling-dependent *ZAM* expression in the posterior-like follicle cells [58]. Therefore, cells where *ZAM* was de-repressed did not seem to have lost their precise differentiation fate. (3) The same was true for the typical antero-posterior gradient of *gypsy*-lacZ de-repression (Fig 6A) that was originally described following specific loss of *gypsy* piRNAs in *flamenco*-permissive mutants [59]. (4) Depletion of individual miRNAs showed that distorted morphology and TE de-repression are two independent phenotypes of miRNA-defective follicle cells. Indeed, we show that no miRNA is involved in these two processes. For instance, oogenesis did not seem to be affected when miR-sponge-mediated titration of either miR-14 or miR-34 resulted in *gypsy*- and *ZAM-*lacZ de-repression.

### The Drosha protein is not directly involved in the piRNA production

Drosha, the RNase III enzyme involved in miRNA biogenesis recognizes and cleaves not only pri-miRNAs, but also many other targets, such as cellular mRNAs [37,60], TEs [61], viral RNAs [62] and long non-coding RNAs [63]. Many Drosha cleavage sites can be folded into local or more long-range secondary structures that could provide the double-stranded substrates preferred by this enzyme. Therefore, we asked whether Drosha could also be directly involved in nuclear processing of the long structured piRNA precursors, in addition to its indirect effect on piRNAs *via* miRNA biogenesis. Our results do not support this possibility (S5 Fig and S4 Table).

In the absence of any evidence for a direct involvement of Drosha endonucleolytic activity in piRNA precursor processing, its crucial role in the biogenesis of at least two miRNAs required for the piRNA pathway integrity remains the most parsimonious way to explain the phenotypes reported here following Drosha activity impairment in follicle cells.

### miR-14 and miR-34 are specifically required for TE piRNA biogenesis and/or stability in follicle cells

In animals, miRNA target recognition is determined by the seed, a short sequence that includes nucleotides 2–8 of the small RNA [64]. The rest of the small RNA matches imperfectly, if at all, to its target. This implies that a single miRNA can target many mRNAs and often operates in highly complex regulatory networks in combination with other miRNAs in the same or different biological processes. This could explain why the ovarian transcriptome is much affected by the global miRNA depletion in follicle cells that express the trans-dominant negative Drosha construct (S7 Table).

In striking contrast with these pleiotropic effects on gene expression, we observed a very specific loss of TE-derived piRNAs with no effect on the accumulation of 3’UTR-derived piRNAs. Indeed, the amounts of piRNA(s) originating from the 3'UTR of genes were not reduced following expression of the trans-dominant negative Drosha mutant, sh*Ago1*, miR-14SP or miR-34SP. They were not affected either in the ΔmiR-14 mutant genetic background (Figs 4, 5, 6D and 6E).

Therefore the piRNA pathway was not impaired at the level of the piRNA producing center that involves Armi, Yb, Shut and the other cytoplasmic proteins known to affect both TE- and 3’UTR-derived piRNAs. Our data suggest that in the case of TEs and the 3’UTR of genes, piRNA biogenesis and/or stability require different actors. Therefore, these piRNAs could follow two somehow separated pathways, at least in follicle cells. More investigations are needed to appreciate to what extent these two somatic piRNA pathways actually differ.

## Materials and Methods

### *WT-Drosha* and *TN-Drosha* plasmid construction

BAC R26A26 (Genbank accession number: AC007084) was digested with *EcoRV* and *NdeI* restriction enzymes, and the resulting 4,5kb fragment containing the *drosha* gene was cloned in the *SmaI/NdeI* restriction sites of the puc19 vector to obtain the *pucWT-Drosha* vector. A Flag-HA tag was introduced at the 3’ end (see S1 Text).

To impair Drosha slicer activity [65], a point mutation in each RNAseIII domain of Drosha was introduced by PCR to produce the *pucTN-Drosha* construct (see S1 Text). *pucWT-Drosha* and *pucTN-Drosha* were digested with *NdeI* and *XbaI* and cloned in the *KpnI* and *XbaI* sites of the pUASp vector. The resulting *pUASp-WT-Drosha* and *pUASp-TN-Drosha* plasmids were introduced in the *w*^*1118*^ strain to get *P-element*-mediated transgenes (BestGene Inc services).

### β**-Gal staining**

Ovaries from 5-day-old flies were dissected in PBS, kept on ice, fixed in 0.2% glutaraldehyde/2% formaldehyde/PBS at room temperature for 5 min and rinsed three times with PBS. They were then incubated in staining solution (1x PBS pH7.5, 1mM MgCl2, 4mM potassium ferricyanide, 4mM potassium ferrocyanide, 1% Triton, 2.7mg/ml X-Gal) at 37°C for either 1h (*gypsy*-lacZ detection) or 4h (*ZAM*-lacZ detection).

### RNA extraction and quantitative RT-PCR

Total RNA was isolated from ovaries with Trizol, following the manufacturer's instructions. RNA was DNase-treated (Turbo DNA-free AM1907, Ambion).

#### mRNA quantification

First strand cDNA was obtained by reverse transcribing 500 ng of total RNA using random primers and SuperScript III (Invitrogen). Quantitative PCR was performed using the LightCycler® 480 SYBR Green I Master system (Roche). Each experiment was performed with biological triplicates and technical duplicates. Relative RNA levels were calculated by using the 2^(-ΔΔCt)^ method [66] and normalized to the appropriate control levels. The RT-PCR primers are listed in S3 Table. Data were analysed with the LightCycler software (Roche).

#### miRNA and piRNA quantification

cDNA synthesis was carried out according to [43] with the following modifications: 100 ng of total RNA was polyadenylated and reverse transcribed in the same reaction tube with E. coli Poly(A) Polymerase (M0276S, NEB) and Superscript II (Invitrogen) and the 5'-CAGGTCCAGT_15_VN primer. Incubation was performed at 37°C for 10 min, then at 42°C for 50 min and finally at 70°C for 15mn (heat inactivation). Primers for quantitative PCR analysis are listed in S3 Table.

### ChIP-PCR

ChIP assay were performed as previously described [67]. Briefly, dissected ovaries were fixed in 1.8% formaldehyde at room temperature for 10 min. Chromatin was sonicated and used for immunoprecipitation with anti-trimethyl-Histone H3 Lys9 (ab8898; Abcam), or anti-dimethyl-Histone H3 Lys4 (ab7766; Abcam) antibodies. DNA precipitates were amplified by real-time quantitative PCR. PCR product levels were normalized to input and expressed relative to a positive control gene (the *1360-element* for the immunoprecipitation with the anti-H3K9me3 antibody and *Rpl15* for the immunoprecipitation with the anti-H3K4me2 antibody). The relative DNA levels were calculated using the following formula: E(target)^CtIP^ *E(ref)^CtInput^ / E(ref)^CtIP^ * E(target)^CtInput^, where E is the efficiency of each primer pair and ref the positive control. Primers are listed in S3 Table.

### Small RNA purification and sequencing

Small RNAs from *tj*-GAL4>Ø, *tj*-GAL4>WT-Drosha* or *tj*-GAL4>TN-Drosha* ovaries (lacking the *tub-Gal80*^ts^ thermo-sensitive Gal4 inhibitor) were isolated on HiTrap Q HP anion exchange columns (GE Healthcare), using the ÄKTA purifier FPLC system as previously described in [67]. The histogram of size distribution and the U1 presence in the small RNA populations sequenced confirmed that this small RNA extraction method efficiently eliminates degradation products (S2 Fig). Small RNAs from *tj*-GAL4>WT-Drosha and *tj*-GAL4>TN-Drosha ovaries were manually isolated on HiTrap Q HP anion exchange columns (GE Healthcare) as described in [68]. Library construction and 50nt read sequencing were performed by Fasteris SA (Switzerland) on an Illumina HiSeq 2000 instrument for the *tj*-GAL4>Ø, *tj*-GAL4>WT-Drosha* and *tj*-GAL4>TN-Drosha* and on an Illumina HiSeq 2500 instrument for the *tj*-GAL4>WT-Drosha and *tj*-GAL4>TN-Drosha libraries.

### Bioinformatic analysis of the sequencing data from the small RNA libraries and RNA-IP samples

Sequencing data were annotated according to the sequences available in several reference databases. rRNA, tRNA and snRNA sequences were retrieved from modEncode (http://www.modencode.org/) [69], miRNA sequences were retrieved from miRBase (http://www.mirbase.org/) [70] and mRNA transcript sequences were retrieved from Flybase (http://flybase.org/). The analysis of small RNA libraries was performed as described in [67]. Briefly, after subtracting the reads matching abundant cellular rRNAs, tRNAs and snRNAs, the remaining reads were considered as *bona fide* small regulatory RNAs reads (siRNAs, miRNAs and piRNAs). miRNAs were separated from the other *bona fide* reads based on their identity with miRBase. Then, piRNAs and siRNAs were identified based on their size (21 nt for siRNAs and 23 to 29nt for piRNAs). piRNA cluster sequences were retrieved according to previously published genomic coordinates [24]. To compare small RNA counts between small RNA-seq samples, libraries were normalized to one million 42AB-derived genome-unique piRNAs (unaffected by *tj*-GAL4>WT-Drosha or TN-Drosha follicle cell-specific expression) (see S4 Table). To compare RNA-IP samples, library read counts were normalized to one million genome-unique reads (see S4 Table).

### RNA-Immunoprecipitation (RNA-IP)

200 ovaries from *tj*-GAL4>Ø, *tj*-GAL4>WT-Drosha and *tj*-GAL4>TN-Drosha flies were dissected in PBS and homogenized in 500μl ice-cold lysis buffer (20mM Tris-HCl pH8, 137mM NaCl, 10% glycerol, 1% Nonidet P40) with complete EDTA-free protease inhibitor (Roche supplier). All further steps were performed at 4°C or on ice. Debris was pelleted at 3 000g for 1 min; supernatants were then collected and pre-cleared with 40 μl mouse IgG-Agarose (Sigma A0919) for 1 h. An aliquot of pre-cleared input was stored. Pre-cleared lysates were immunoprecipitated with anti-FLAG M2 affinity gel (Sigma A2220) at 4°C overnight. The anti-FLAG M2 affinity gel was washed four times with lysis buffer and the precipitated complexes were eluted with 200ng/μl 3X FLAG Peptide (Sigma F4799) in lysis buffer. Eluates were then immunoprecipitated with the anti-HA antibody (Santa-Cruz SC805) coupled with Dynabeads protein G (Invitrogen 10004D). Immunoprecipitates were washed four times with lysis buffer. RNA from inputs and immunoprecipitates was extracted with TRIzol, rRNA-depleted using the RiboMinus Eukaryote Kit for RNA-sequencing (Invitrogen) and DNAse-treated (Turbo DNA-free AM1907, Ambion).

Samples were then processed and sequenced by Fasteris SA (Switzerland). Briefly, RNAs were fragmented (zinc treatment, Illumina protocol), reverse transcribed with random hexamer primers and 260 to 280 bp fragments (i.e., insert of 160–240 nt) were purified on acrylamide gels. Reads from 50nt were sequenced on HiSeq2000 (Illumina). The RNA orientation was ignored in these experiments.

### Statistical analyses

All statistical analyses were performed using the SciPy library (http://scipy.jp/scipylib/citing.html). *P*-values were calculated using two-tailed Student’s *t*-tests for samples displaying normal distribution (tested with the Shapiro-Wilk test). The variance homogeneity was tested with the Levene’s test. When at least one of the two series did not have a normal distribution, *P*-values were calculated using the Mann-Whitney rank-sum test (with correction for continuity).

### GO term analysis

GO analysis was performed using the Gorilla [71][72] online tool (http://cbl-gorilla.cs.technion.ac.il/) and two ranked lists of genes. The background list consisted in all the genes targeted by miRNA families in *Drosophila melanogaster* (taxon id 7227) given by TargetScanFly (http://www.targetscan.org/fly_12/fly_12_data_download/Conserved_Family_Conserved_Targets_Info.txt.zip). The adjusted P-values were corrected for multiple testing using the Benjamini-Hochberg method.

### Accession numbers

Small RNA data from Yb heterozygous and mutant ovaries were previously published (Handler *et al*. Embo J. 30:3977) and are available *via* the NCBI Gene Expression Omnibus (accession no. GSM767598 and GSM767599 respectively). Sequencing data concerning the small RNA and RNA-IP libraries generated in this study are available via the NCBI Gene Expression Omnibus (GEO) (http://www.ncbi.nlm.nih.gov/geo/) under accession no. GSE60974 (see S4 Table for details).

## Supporting Information

S1 Fig Validation of the trans-dominant negative strategy and of the *drosha* RNAi approach. *(A*) Schematic representation of the domain organization of Flag-HA-tagged wild type (WT) and trans-dominant negative (TN) Drosha constructs. In the TN-Drosha construct, each RNAseIII catalytic domain was inactivated by substituting an essential Asp residue with an Ala residue. dsRBD: double-stranded RNA binding domain. *(B*) Co-immunoprecipitation of endogenous Pasha (a Dosha-binding protein) and *tj*-driven Flag-HA-tagged WT- and TN-Drosha proteins. Ovaries containing only the *tj-Gal4* driver (*tj*-GAL4>Ø; no Flag-tagged protein) were used as controls of the anti-Flag immunoprecipitation (middle). *(C*) *tj-*driven expression of TN-Drosha or of the two long *Drosha* inverted repeats (*drosha-IR*) in follicle cells resulted in stabilization of the three tested pri-miRNAs. Quantification was done relative to *RpL32* and normalized to the respective controls (Ø>*drosha-IR* for *tj*-GAL4>*drosha-IR* and *tj*-GAL4>Ø for both *tj*-GAL4>WT-Drosha and *tj*-GAL4>TN-Drosha) (bars represent the mean ± SD, n = three biological replicates, log2 scale). (*D*) *tj-*driven expression of TN-Drosha in follicle cells correlates with the loss of the three tested miRNAs. The miRNA level was assayed using follicle cell-enriched ovarian extracts. The miRNA level was expressed relatively to a follicle cell-specific RNA (*tj*) and normalized to control (*tj*-GAL4>WT-Drosha) (bars represent the mean ± SD of three biological replicates).
(EPS)

S2 Fig General profile of *tj*-GAL4>WT-Drosha and *tj*-GAL4>TN-Drosha small RNA-seq libraries. (A) Barplots displaying the length distributions of *tj*-GAL4>WT-Drosha* (left panel) and *tj*-GAL4>TN-Drosha* (right panel) mapper reads normalized to one million of genome-unique 42AB mappers. (B) WebLogo on mapper reads of both *tj*-GAL4>WT-Drosha* (left panel) and *tj*-GAL4>TN-Drosha* (right panel) libraries. The logo was build using WebLogo software (http://weblogo.berkeley.edu/). The height of each letter represents the relative proportion of each nucleotide at each position.
(EPS)

S3 Fig Confirmation of piRNA loss in follicle cells expressing trans-dominant negative Drosha (related to Fig 2). (A-B) Normalized profiles of ovarian piRNAs (sense: up (red); antisense: down (blue)) mapping to TE consensus sequences (four mismatches allowed) and to piRNA clusters in *tj*-GAL4>WT-Drosha (upper panels) and *tj*-GAL4>TN-Drosha (lower panels) libraries. Libraries were normalized to 1 million of genome-unique 42AB mappers, a germline-specific piRNA cluster. The y axis indicates the number of piRNAs collapsed to their 5’ end. (A) Profiles of piRNAs mapping to the *F-element* (a germline-specific TE; left), or to *ZAM* (middle) and *Tabor* (right) (two follicle cell-specific TEs). (B) Profiles of piRNAs originating from a germline-specific piRNA cluster (80 E-F), two follicle cell-specific piRNA clusters (*flamenco* and cluster #17). Only genome-unique piRNAs are mapped.
(EPS)

S4 Fig De-repression of the ZAM follicle cell-specific TE in ΔmiR-14 null mutant ovaries (related to Fig 6). Fold changes in the steady-state RNA levels of *ZAM*, *Tabor* and *Rpt5* (negative control) induced by the ΔmiR-14 null mutation. Quantification was done relative to *RpL32* and normalized to the heterozygous siblings (error bars represent the SD of three biological replicates). Note that, since the heterozygous ΔmiR-14/Cy control might contain more TE copies than the mutant (because Cy is very likely to be a TE-rich balancer chromosome), TE de-repression could have been under-estimated.
(EPS)

S5 Fig Drosha is not involved in the nuclear cleavage of *flamenco* piRNA cluster transcripts. Fold changes in the steady-state RNA levels of five short (region 1 to region 5: about 100 nt-long) and three long (extended region 1 to extended region 3: 300–400 nt-long) fragments from the *flamenco* piRNA cluster (see S3 Table for primer sequences) upon expression of the trans-dominant negative Drosha mutant (TN-Drosha). Quantification was done relative to *RpL32* and normalized to the tj-GAL4 driver (tj>GAL4>Ø) (error bars represent the SD of three biological replicates).
(EPS)

S6 Fig *ZAM*-lacZ maintains its idiosyncratic tissue-specific expression pattern even in distorted miRNA-defective ovaries. Shown is the β-Gal staining of ovaries subjected to constitutive (larval, pupal and adult development at 28°C) *tj*-driven expression of either WT- or TN-Drosha. Blue arrows point towards the β-Gal staining of "polar-like" follicle cells facing a mislocalized oocyte, identified by its darker vitellus.
(EPS)

S1 Table List of the Drosophila stocks used in this study. (XLS)

S2 Table Detailed genotype of the ovaries used in the different figures. (XLSX)

S3 Table Primer list. (XLS)

S4 Table Library annotation. (A) Sequencing counts for all generated small RNA libraries. “Mappers” correspond to reads perfectly mapping to the *Drosophila melanogaster* sequenced genome. miRNA reads were separated from the other small regulatory RNAs read based on their identity with miRBase (http://www.mirbase.org/). Then, piRNAs and siRNAs were identified based on their size (21 nt for siRNAs and 23 to 29 nt for piRNAs). The 42AB reads correspond to 42AB-derived genome-unique piRNAs and the *flamenco* reads correspond to *flamenco*-derived genome-unique piRNAs. (B) Sequencing counts for all generated RNA-IP samples. “Mappers” correspond to reads perfectly mapping to the *Drosophila melanogaster* sequenced genome. Pri-miRNA reads were annotated based on their identity with miRBase (http://www.mirbase.org/). *Flamenco* mapped reads correspond to reads that can derive from *flamenco* whereas the *flamenco* uniquely mapped reads correspond to reads exclusively coming from *flamenco*.
(XLS)

S5 Table Normalized number of reads for each of the 53 miRNAs co-immunoprecipitated with TN-Drosha protein expressed in follicle cells. The number of RNA reads sequenced in the three inputs (input (*tj*-GAL4> Ø, tj-GAL4>WT-Drosha and *tj-*GAL4>TN-Drosha)) and in the three IP experiments were normalized to one million genome-unique reads. ND indicates that these six miRNAs have not been tested in the miR-SP genetic screen.
(XLS)

S6 Table List of miR-14 putative target genes enriched in the GO terms GO0016020 and GO0030139. (XLS)

S7 Table Fold change in mRNA levels between *tj*-GAL4>WT-Drosha and *tj*-GAL4>TN-Drosha. The number of reads in each input was normalized to one million genome-unique reads.
(XLS)

S1 Text Supporting Materials and Methods. (DOC)
