# Supplementary material for: MicroRNA-Dependent Transcriptional Silencing of Transposable Elements in Drosophila Follicle Cells
Source: PLoS Genet. 2015 May 19;11(5):e1005194. doi: 10.1371/journal.pgen.1005194 (PMC4451950; doi:10.1371/journal.pgen.1005194)
Supplement: S1 Text — (DOC) [file pgen.1005194.s014.doc]

Supporting Materials and Methods

**Construction of the *pUASp-*WT-Drosha and *pUASp-*TN-Drosha vectors**

The primers used to introduce the Flag-HA epitope at the 3’end were: 5' gtgtcttcaaaatcttcgCTGGGAgactacaaggacgacgatgacaagCT

Agcggccgcaggata 3' and 5'cgcaaagctgttcatcCCAGggcgtagtcgggcacgtcgtaggggtatcctgcggccgcTAGct 3'. The two primers were annealed and the Klenow enzyme was used to obtain a blunt, double-stranded DNA fragment. This fragment was restricted with *Bse*YI and cloned into the *pucWT-Drosha* vector partially restricted with *Bse*YI.

*pUASp-TN-drosha* was obtained by four consecutive PCR amplifications performed using the tagged *pucWT-Drosha* plasmid with the following primer pairs:

| oligonucleotide sequences | PCR product name |
| --- | --- |
| 5'ctccgccagaagaagatctatctccacc  5'cagcaGcaccgaggaactcGagtctctcattgtgg | D1 |
| 5' gagagactCgagttcctcggtgCtgctgtagtagaattcctcagc  5' cactgtggcgcccaagaattctaaacgctg | D2 |
| 5' atgctgtacgcacacggatccgatc  5' cactgtggcgcccaagaattctaaacgctg | D3 |
| 5' gtttagaattcttgggcgccacagtgctgc  5'tatcctgcggccgctagcttgtcatcgtcgtccttgtagtctcccag  cgaagattttgaagacac | D4 |

(mutated bases are in capital letters)

The D1 and D2 PCR products were restriction enzyme-digested with *Xho*I and ligated together to obtain the D5 DNA fragment of 3063bp. D5 harbours an A to C substitution at position 3059 leading to the replacement of an aspartic acid by an alanine in the first RNAseIII domain of Drosha.

D3 was digested with *Eco*RI and D4 partially digested with *Eco*RI. These two fragments were then ligated together to obtain a 1422 bp fragment named D6. D6 contains an A to C substitution at position 392 leading to the replacement of an aspartic acid by an alanine in the second RNAseIII domain of Drosha.

D5 and D6 were then digested with *Bam*HI and ligated together to obtain D7, a double stranded DNA of 4086 bp. D7 was digested with *Mlu*I and *Nhe*I and the resulting 2229 bp fragment was subcloned into the same restriction sites of the tagged *pucWT-Drosha* construct to obtain *pucTN-*Drosha. This last construct was sequenced to ensure that no unplanned mutation was introduced with the PCR amplifications.

### Immunoprecipitation and western blot analysis

50 ovaries were dissected in PBS and homogenized in 300µl of ice-cold lysis buffer (20mM Tris-HCl pH8, 137mM NaCl, 10% glycerol, 1% Nonidet P40) with complete EDTA-free protease inhibitor (Roche). All further steps were performed at 4°C or on ice. Debris was pelleted at 3 000g for 1 min; supernatants were collected and pre-cleared with 40 µl mouse IgG-Agarose (Sigma A0919) for 1 h. An aliquot of pre-cleared input was saved for protein analysis (20 µl). Pre-cleared lysates were immunoprecipitated with anti-FLAG M2 affinity gel (Sigma A2220) at 4°C overnight. An aliquot of each supernatant was saved for protein analysis (20 µl). Anti-FLAG M2 affinity gel was washed four times with lysis buffer. The precipitated complexes were eluted with 3X FLAG Peptide (Sigma F4799) at a concentration of 200ng/µl and used for western blot analysis. For western blotting, the following primary antibodies were used: rabbit anti-HA (sc-805; 1:5000), anti-Pasha (monoclonal 1E3-C; 1:50) and anti-Loqs (monoclonal 1E4: 1:5000). Anti-rabbit or -mouse secondary antibodies were horseradish peroxidase conjugated (Pierce); antibody interactions were detected by Enhanced ChemiLuminescence (Supersignal West Femto from Pierce).

**RT-PCR to detect pri-miRNAs**

The experimental conditions for RT-PCR are described in Materials and Methods. The primers used to detect the pri-miRNA are listed in Table S3.

**Preparation of follicle cells.**

Follicle cells were isolated as described in Bryant et al., (1999) *P.N.A.S.,* 96:5559, with the following modifications: cells were washed in PBS and lysed in TRIzol.
